# Seasonal phoresy as an overwintering strategy of a phytophagous mite

**DOI:** 10.1038/srep25483

**Published:** 2016-05-06

**Authors:** Sai Liu, Jianling Li, Kun Guo, Haili Qiao, Rong Xu, Jianmin Chen, Changqing Xu, Jun Chen

**Affiliations:** 1 Institute of Medicinal Plant Development, Chinese Academy of Medical Sciences & Peking Union Medical College, Beijing 100193, China

## Abstract

Migration by attachment to insects is common among mites that live in temporary habitats. However, because plants provide relatively stable habitats, phytophagous mites are generally not dependent on other animals for dispersal, so whether these mites can consistently be phoretic on insects through a particular life stage remains unclear and controversial. Here, we describe an obligate phoresy of a wholly phytophagous mite, *Aceria pallida*, in which the mites accompanied the psyllid *Bactericera gobica* to its winter hibernation sites, thus successfully escaping unfavourable winter conditions, and returned to reach the buds of their host plant early the following spring. This finding provides evidence of a new overwintering strategy that has contributed to the evolutionary success of these tiny phytophagous mites.

Winter is a difficult time for all arthropods because these animals are exposed to extreme physical conditions, so locating an overwintering site is often critical to their survival^1^,^2^. Unlike winged insects^3^,^4^, searching for a suitable overwintering site is more difficult for tiny and wingless mites, which generally have limited mobility^5^,^6^. However, some parasitic or phoretic mites can attach themselves to larger carriers (such as the parasitic bee mite *Acarapis woodi* Rennie^7^ and the phoretic bumblebee mite *Parasitellus fucorum* De Geer^8^) and accompany them to their hibernation sites for overwintering^7^,^8^. However, this behaviour has not yet been reported in phytophagous mites^6^,^9^,^10^,^11^; almost all phytophagous mites overwinter on the host plant or in the soil under the host plant canopy^6^,^9^,^10^,^12^,^13^,^14^.

Phoresy is a common phenomenon among arthropods, in which an organism attaches itself for a limited time period to another during migration from the natal habitat to a new and potentially better habitat^15^,^16^,^17^. The most common phoretic associations occur among the insects and mites that live in temporary habitats^16^,^18^,^19^, such as some dung- and carcass-visiting insects, especially flies (such as *Muscina stabulans* Fall. and its phoretic mite *Myianoetus muscarum* L.)^18^,^20^ and burying beetles (such as *Nicrophorus vespilloides* Herbst and its phoretic mite *Poecilochirus carabi* G. Canestrini & R. Canestrini)^18^,^21^,^22^. However, because such plants provide stable rather than temporary micro-habitats, little information about phoresy as a migration strategy in wholly phytophagous mites has been reported^5^,^6^,^9^,^12^,^23^. Although some mites (eriophyoid, tetranychid, and phytoseiid mites) may rarely be found on insects^5^,^24^, this appears to be a fortuitous or secondary phenomena^5^,^14^, and these mites do not exhibit obligate phoresy.

Eriophyoid mites are the smallest of all of the plant feeders and are typically less than 200 μ m in length^25^. They have a worm-like body with only two pairs of legs and tend to be highly host specific or even tissue specific^11^,^25^. These mites tend to overwinter as adult females in diapause form (deutogynes) or as eggs on their host plant, and they are most frequently found on buds and in bark crevices^13^,^23^,^26^. Because of their host specificity and limited mobility, eriophyoid mites seem to disperse by attaching to other animals, which has been reported by several authors^5^,^27^, and while this has often been interpreted as phoresy, the results are experimentally limited and remain equivocal and controversial^5^,^27^,^28^.

Additionally, some acarologists have concluded that all uses of carriers are accidental because eriophyoid mites do not show clear phoretic morphological adaptations (pedicels or claws) or specific adaptations for selectivity of more favourable carriers^6^,^14^,^16^,^28^. Furthermore, research on phoresy in phytophagous mites has mostly focused on the protonymphs during growing seasons, but most mite phoresy occurs in deutonymphs when an ephemeral habitat degrades or disappears^16^,^29^.

The gall mite *Aceria pallida* Keifer (Acari: Eriophyidae) and the psyllid *Bactericera gobica* Loginova (Hemiptera: Psyllidae) are the most important pests of the wolfberry^30^, *Lycium barbarum* L. (Solanaceae), which is widely cultivated in northwestern China and is of great importance in Chinese traditional medicine^31^,^32^. Preliminary observations found no mites in their common hibernation sites on the host plant, but many mite galls were found in spring buds and were closely related to the eggs laid by psyllids. We hypothesized that these psyllids likely contribute to the breakout and prevalence of the mites because no galls occurred if the buds were isolated from adult psyllids in early spring. Therefore, we postulated that mite phoresis on psyllids is not accidental.

Thus, we investigated this mite phoresis on psyllids over the course of 2 years, focusing on the attachment period in the late fall and the detachment period in the early spring, and the adaptive phoretic structure was also identified. The primary hibernation sites of the mites were examined, and the artificial induction of phoresy and detachment was studied.

## Results

### Seasonal phoresy of *A. pallida*

**Figure 1:**
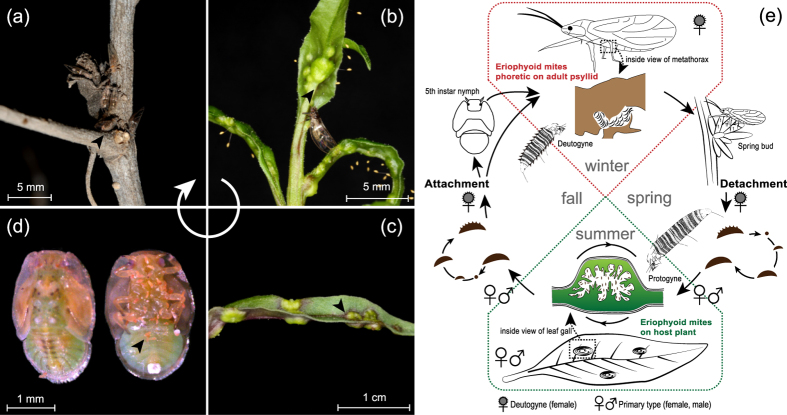
Photographs and life history diagram showing the phoretic association between eriophyoid mites and psyllids. (**a**) Phoretic mites associated with adult psyllids during the winter. (**b**) Phoretic mites dismounted from psyllids and forming galls on the host plant in early spring. (**c**) The galls on the host plant caused by eriophyoid mites in summer. (**d**) Phoretic mites hidden under the abdomen of a 5th instar psyllid nymph in autumn. (**e**) The life history of an eriophyoid mite that can be phoretic on a psyllid and accompany the host during the hibernation period.

The life history of *A. pallida* is shown in Fig. 1. In winter, the activity of the phoretic mites on the psyllids almost completely ceases, but the psyllids may seek a comfortable site to protect themselves from harsh conditions (Fig. 1a). Thus, these insects represent a favourable environment for phoretic mites. As the temperature increases in early spring, the psyllids immediately feed on the buds of the host plant, and their activities (feeding, mating and laying eggs) provide the mites sufficient time to dismount (Fig. 1b). Afterward, the mites reproduce and live inside the galls of the host plant (Fig. 1c), and their dispersal could be by wind and other means in spring and summer. In late autumn, the mites emerge from the galls and temporarily hibernate under the abdomen of the inactive psyllid nymphs (Fig. 1d) and then transfer to the adults during or after eclosion. This strategy constitutes a well-developed, obligate seasonal phoresy that allows these eriophyoid mites to survive winter by attaching to the psyllids and to be transported to the host plant in the early spring (Fig. 1e).

**Figure 2:**
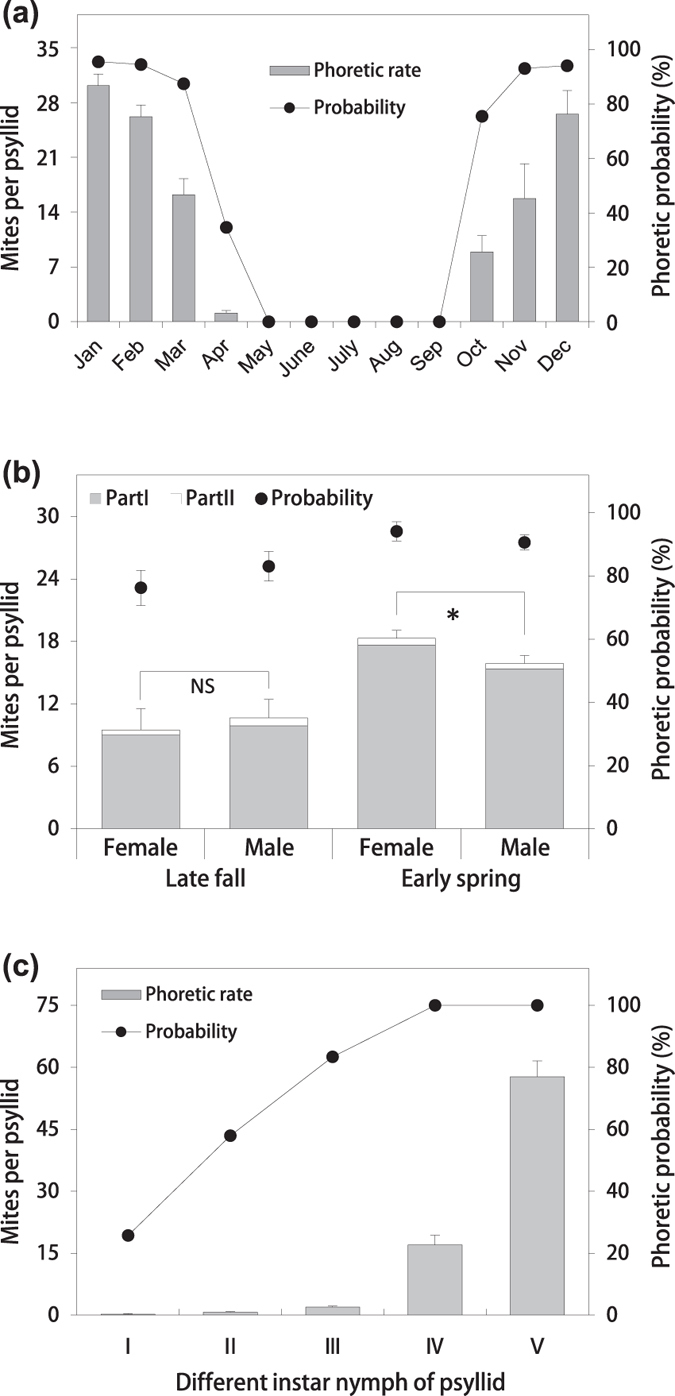
(**a**) A monthly time series showing the phoretic probability (the primary *y*-axis; solid line and circles) and the phoretic rate (the secondary y-axis; grey columns) of *A. pallida* throughout the season. Error bars are + SE. (**b**) The phoretic rate and phoretic probability of *A. pallida* on females and male *B. gobica* adults in the late fall (59 females and 65 males; t =  − 0.433, df =  122, P =  0.664, which is > 0.05) and in the early spring (480 females and 407 males; t =  2.26, df =  885, P =  0.024, which is < 0.05). For Part I and Part II, see figure 4a. Asterisks denote a significant difference between adult females and males of *B. gobica* (*P <  0.05), and “NS” denotes no significant difference. (**c**) The phoretic rate and phoretic probability of *A. pallida* on different psyllid nymph instars.

The annual prevalence and phoretic rate of the mites on the psyllids (2 years of data) are shown in Fig. 2a, and both decreased from March (87%, 16.35 ±  2.05) to April (34%, 1.1 ±  0.32) during the spring detachment period (Fig. 1b,e). No phoretic mites were found on the adult psyllids, except for sporadic mounting on the body surface and wings, during the vigorous *L. barbarum* growth period from May to September. Nevertheless, as the temperature decreased and the leaves fell, the prevalence and phoretic rate increased rapidly from October (75%, 8.98 ±  2.10) to November (93%, 15.9 ±  4.47) and reached a steady state in December (93%, 26.7 ±  3.04) (Fig. 1a,e).

The phoretic probability did not differ significantly between the sexes of the psyllids in late fall (female: 76%, n =  59; male: 83%, n =  65; t =  − 0.94, P =  0.35) or early spring (female: 94%, n =  480; male: 91%, n =  407; t =  1.95, P =  0.052) (Fig. 2b). In the late fall, there was also no significant difference in the phoretic rate between the two sexes (female: 9.0 ±  2.0; male: 9.89 ±  1.78; t =  − 0.433, P =  0.664), while during the phoretic steady-state period in the early spring, there were more phoretic mites on the female psyllids than on the males (female: 18.63 ±  0.75; male: 15.52 ±  0.75; t =  2.26, P =  0.024, which is < 0.05).

During the autumn attachment period, when the deutogynes of *A. pallida* emerge from the galls, the mites could be exposed to unfavourable conditions, so they temporarily hibernate under the abdomens of the inactive psyllid nymphs and then attach themselves to the adults during or after eclosion (Fig. 1d,e). Additionally, the phoretic rate and probability increased as the nymph instars of *B. gobica* developed (1st: 0.29, 26%; 2nd: 0.73, 58%; 3rd: 1.97, 83%; 4th: 17.03, 100%; 5th: 57.71, 100%) (Fig. 2c), and the maximum phoretic rate on the 5th instar nymph was 117.

### The primary hibernation site of *A. pallida* is not on the host plant but as a phoretic on the psyllids

**Figure 3:**
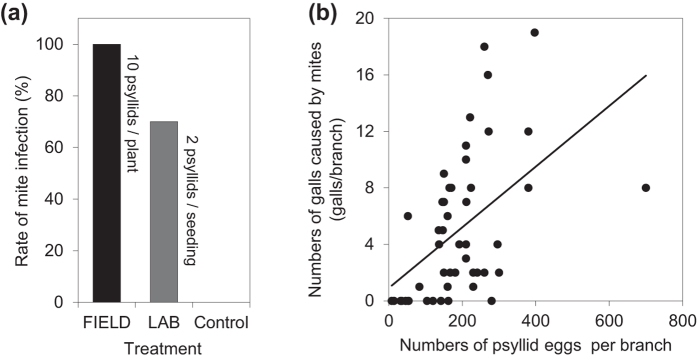
(**a**) Rate of mite infection of the host plant under different treatments. (**b**) Regression of the observed numbers of galls caused by mites against the numbers of psyllid eggs (reflects the time spent on the psyllid) on the host plants (n =  20). Galls and eggs were counted after adult psyllids completed oviposition and died on 27 April.

No *A. pallida* gall mites were found surviving in the typical hibernation sites (bark, branches and buds)^13^,^33^,^34^, but live deutogynes of another free-living eriophyoid mite, the wolfberry rust mite *Aculops lycii* Kuang, was observed, although none were present on the psyllids. Moreover, the galls caused by *A. pallida* were not found on the plants that were isolated from the psyllids, but the mite infection rate was 100% when 10 adult psyllids were released on each plant (Fig. 3a). Furthermore, the number of galls caused by detached mites was significantly correlated with the number of psyllid eggs (P <  0.001, Fig. 3b); more eggs imply that the adult psyllids spend more time on these leaves and provide more time for the mites to detach.

### Phoresy and dismount induction

#### Phoresy induction

The phoresy of *A. pallida* was also artificially induced under conditions similar to the attachment period in late autumn, with low and alternating day-night temperatures (day 8 h: 15 °C; night 16 h: 10 °C for 4 h, 5 °C for 8 h, 10 °C for 4 h) and a short photoperiod (30–60% RH, 8: 16 h L: D). Phoretic behaviour by the mites was first observed after 21 days, and the phoresy probability reached 80% after 28 days. In the control treatment (25 °C, 30–60% RH, 16: 8 h L: D), non-phoretic behaviour on the psyllids was observed throughout the experiment.

#### Dismount induction

The phoretic mite *A. pallida* can easily dismount from its carrier psyllid to the host plant under suitable conditions (25 °C, 30–60% RH, 16: 8 h L: D), and the mite infection rate was 70% after only 5 days when 2 psyllids were released per seedling (Fig. 3a).

### The entire worm-like body of *A. pallida* is structurally adapted for phoresy

**Figure 4:**
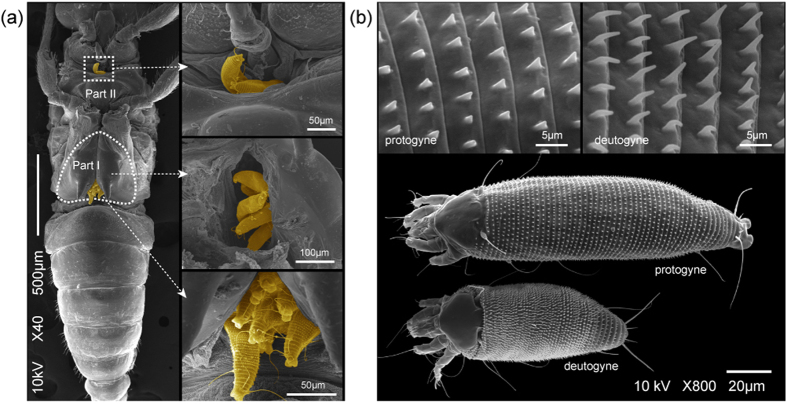
(**a**) Deutogynes of *A. pallida* (orange) phoretic on adult *B. gobica*; the locations of harbouring sites are indicated by the squares drawn in dotted lines. Part I: the space under the inside of both metapedes coxae on the metathorax; Part II: the space near the rostrum. (**b**) The top view and microtubercles of the *A. pallida* protogyne and deutogyne, which are indicated in the picture.

There were two structures on the body of *B. gobica* suitable for mite attachment (Fig. 4a). Part I was the space under both metapedes coxae on the metathorax, and Part II was adjacent to the rostrum. These two structures include some spaces, which are surrounded by inter-segmental membranes, in which the vermiform mites can conceal themselves. More than 97% of the mites were phoretic on Part I of the psyllids (Fig. 2b), with up to 72 phoretic mites on female psyllids and 67 on male psyllids, and *A. pallida* can also attach to other psyllids with similar structures, such as *E. robinae* (phoretic rate: 7.46 ±  1.81, phoretic probability: 73%). Additionally, the mites can also occasionally be found on other arthropods (ants, aphids, ladybugs, stinkbugs, and beetles), but none have structures similar to those of the psyllids to facilitate phoresis by the mites.

With the exception of the length of the proterosoma (P =  0.220) (Table 1, Fig. 4b), the length of the hysterosoma of the deutogynes (128.34 ±  14.55 μ m) was approximately 1/2 the size that of the protogynes (207.27 ±  22.45 μ m) (P <  0.001), and the width of the deutogynes (47.26 ±  3.49 μ m) decreased to 7/10 of the protogynes (67.56 ±  11.1 μ m) (P <  0.001). However, the length of the deutogyne microtubercle (1.59 ±  0.18 μ m) was 50% longer than that of the protogynes (1.02 ±  0.17 μ m) (P <  0.001).

## Discussion

Phoresy among arthropods as a means of migration from degraded habitats is ubiquitous in nature^16^,^18^,^29^, but phoresy as an overwintering strategy in phytophagous arthropods seems to be much rarer. In this study, we observed that wholly phytophagous gall mites, the deutogynes of *A. pallida*, have evolved a suite of complementary behavioural and structural characters in response to the seasonal challenge of overwintering and locating suitable habitats on host plants in early spring. Because they share the same host plant and degree of host specificity as the psyllid *B. gobica*^35^, the mites can dismount when the carrier insects arrive at the host plant and then reproduce on it.

We suggest that this novel overwintering strategy may have evolved from the shelter-seeking behaviour of the phytophagous mites under cold and dry environmental conditions^10^,^26^. Mites that live in shelters (gall-inducing and refuge-seeking species) may be more likely to be phoretic on arthropods than those that are free-living. Based on our observation that no rust mites, *A. lycii*, were found on *B. gobica* during the hibernation period, we deduced that of the rust mite *A. lycii* and the gall mite *A. pallida*, two eriophyoid species on the same host plant *L. barbarum*, only the gall-forming mite is phoretic on psyllids as an overwintering strategy. This could be explained by the different lifestyle and morphological characteristics of the species: free-living eriophyoid mites may have a relatively solid prodorsal shield and thicker tergites for resistance to winter conditions than the shelter-seeking mites^28^. Furthermore, the harsh winter conditions, the presence of a suitable carrier on the same host plant, the life history synchronization with the host, and particularly, the structural adaptations of *A. pallida* (such as its worm-like body and the appropriate space under both metapedes coxae of *B. gobica*) are likely the essential factors that promoted the evolution of this relationship.

In relation to the phoretic host (*B. gobica*) or other psyllids (such as *E. robinae*), the worm-like body of the deutogyne of the eriophyoid mite (*A. pallida*), with its accentuated development of microtubercles but shortened body size, constitute a special structural adaptation for phoresy, and we suggest that the structure and stability of these morphological adaptations are not inferior to those of other phoretic mites. In addition, the accentuated development of the microtubercles in the eriophyoid mites, which facilitate forward movement in confined spaces, is analogous to the function of the chaetae of annelid worms^28^. The deutogynes of the eriophyoid mites generally exhibit reduced or suppressed microtuberculation, which seems to conserve the body fluids of hibernating deutogynes, rendering their cuticles more resistant to water loss^28^. The wolfberry rust mite, *A. lycii*, which overwinters on the host plant, has similar structural features (electronic supplementary material, Fig. S1). However, the microtuberculation of the *A. pallida* deutogynes are longer than those of the protogynes, so we suggest that the carrier insect may provide a relatively humid environment for this mite during hibernation, and the need for microtuberculation to achieve phoresis may have driven the development of this structural difference.

The phoretic association between the mites and insects^16^,^20^,^36^ and the association between the gall-forming arthropods and the host plant^37^,^38^,^39^,^40^,^41^ have been considered to be model systems for studies of coevolution, but the phoresy of the gall mite may indicate a more advanced and complicated association between the mite, the carrier insect and the host plant. Because gall-forming arthropods are well known for their ability to manipulate host-plant morphology and physiology^37^,^39^, they could affect the performance of other herbivores. For example, the gall mite *Aceria cladophthirus* Nalepa increases the susceptibility of its host plant to spider mite *Tetranychus urticae* Koch^42^, and the performance of a butterfly *Neuroterus saltatorius* Edwards decreases with increasing gall wasp *Erynnis propertius* Scudder & Burgess density^43^. Furthermore, the galls caused by the midge *Rabdophaga salicisbrassicoides* Packard increase the abundance of aphids and their attendant ants^44^. The gall mite *A. pallida* can undoubtedly benefit from phoresy, but whether the mites benefit their carrier psyllids in direct or indirect ways during the growing season and whether they are clearly associated throughout the year require further study.

Our results help to settle the debate regarding the existence of phoresy in phytophagous mites^5^,^12^,^14^,^28^ because the seasonal phoresy of *A. pallida*, based on the definition and classification^15^,^16^,^17^, is a typical obligate phoresy. Although many phytophagous mites have some adaptations for dispersal by other carriers in the growing season^5^,^14^,^24^, they are better adapted to expand their habitats than to overcome harsh conditions. Michalska and Skoracka^5^ have summarized the different dispersal modes of eriophyoid mites (wind, carriers, ambulation, and rain), but almost all of these dispersal mechanisms occur in the growing season, and none of the dispersal modes by carriers can be considered a type of phoresy. Thus, we deduced that the dispersal in the growing season should be understood as a normal population dynamic event, while phoresy as a migration strategy in phytophagous mites represents a seasonal displacement of a population^45^.

Another interesting finding is that the number of phoretic mites on *B. gobica* females was higher than on males following a harsh winter, but we suggest that the mites cannot discriminate the between sexes or even among species (such as *E. robinae*) of psyllid hosts. Instead, the adult female psyllids always have a larger body size^35^ and a longer lifespan^46^ that may provide more space for passengers and enable the mites to survive for a longer time despite difficult winter conditions. Furthermore, we suggest that female psyllids should be better hosts for phoresy because the adult females will spend more time on the buds of the host plant in early spring when laying their eggs.

We suggest that the phoresy of other phytophagous mite species may have evolved similarly to that reported here for *A. pallida*, and because these phoretic phytophagous mites may have the potential to be seriously harmful pests, their control and quarantine should be achieved by eliminating their carrier insects. Furthermore, when investigating the phoresy of other phytophagous mites, more attention should be paid to the insects and other arthropods that share the same habitat during the hibernation period. Finally, the gall-inducing mites on deciduous plants, especially the species in the *Aceria* genus that share the same habitat as the psyllids, may have greater potential for phoresy and require further study.

## Methods

### Investigations of phoretic mites

All field experiments were conducted in Zhongning County (N37°28′ , E105°42′ ) in Ningxia province, China, and the routine investigation of the monthly phoretic rate was conducted at the experimental field station from January 2013 to December 2014. The number of psyllids sampled monthly from January to December were 90, 90, 951, 124, 103, 100, 90, 90, 90, 240, 60 and 60, respectively. The investigation of the loading period of the eriophyoid mites onto the psyllids was performed from 1 to 21 October 2013 (n =  124), and we simultaneously investigated the phoretic rates of different *B. gobica* nymph instars (n =  35 per instar). The phoretic mites in the steady-state period were investigated from 1 to 12 March 2014 (n =  887) at 8 different sites located near the cities of Zhongning (N37°29′ 51″ , E105°41′ 33″; N37°32′ 41″, E105°37′ 53″; N37°28′ 55″, E105°42′ 48″; N37°30′ 9″, E105°37′ 42″; N37°28′ 13″, E105°37′ 27″; N37°28′ 57″, E105°42′ 47″; N38°55′ 22″, E103°21′ 0″; and N38°43′ 36″, E103°0′ 37″).

The adult psyllids were collected from the host plant using an aspirator (11 cm in height and 3 cm in diameter) and transferred to the lab. The carriers (*B. gobica*) were fastened onto stubs with double-sided tape, and a fine, bent micropin was used to slit the two large hind coxae from the midline; the sexes of the psyllids and the phoretic mites on different parts of the carriers were recorded under a Leica M205C stereomicroscope (Leica Microsystems, Wetzlar, Germany). The phoretic rate was expressed as the number of mites attached per psyllid, and the phoretic probability was expressed as the ratio of the number of psyllids that carried mites to the total number of psyllids.

### The verification of the primary overwintering sites

There were two eriophyoid species (the gall mite, *A. pallida*, and the rust mite, *A. lycii*) on the same host plant (*L. barbarum*), and we verified the presence of the deutogyne mites of these two species in their general hibernation sites (bark, n =  500; branches, n =  800; buds, n =  1200)^13^,^33^,^34^ at the experimental site from 10 to 12 March 2014. Simultaneously, we isolated 40 wolfberry trees (1 m in height, 2 cm in stem diameter, 2 years old, and infected with *A. pallida* during the last year) with a 60-mesh net (1.2 m in height and 0.8 m in diameter). We tapped the branches and the stem with a crabstick to drive the psyllids away, cleaned the litter layer and ensured that all of the psyllids were clear before isolation. The experimental treatment consisted of the release of 10 adult psyllids (5 females and 5 males) inside the mesh net (n =  20), and the control treatment was isolated from the psyllids (n =  20). On 25 April 2014, the number of galls caused by *A. pallida* and the number of eggs laid by *B. gobica* on the leaves approximately 20 cm from tip of the branch were recorded. The branches (n =  48) were randomly selected from the isolated trees.

### Phoresy and dismount induction

All plants (*L. barbarum*) used in the experiments were grown from seeds in plastic pots (9-cm high and 6-cm wide) in a climate-controlled room (25 °C, 30–60% RH, 16: 8 h L: D). Clean plants (i.e., free of eriophyoid mites, psyllids and damage) were used as the experimental materials. Stock cultures of *A. pallida* and *B. gobica* were started from individuals collected at the experimental site in March 2013 and were cultured on wolfberry seedlings in a climate-controlled room (25 °C, 30–60% RH, 16: 8 h L: D).

#### Phoresy induction

On 23 June 2014, the wolfberry plants (n =  32) that were infected with *A. pallida* (1 month old) under “suitable” conditions (25 °C, 30–60% RH, 16: 8 h L: D) were transferred into a climate-controlled box under “unsuitable” conditions similar to the attachment period in late autumn, with low and alternating day-night temperatures (day 8 h: 15 °C; night 16 h: 10 °C for 4 h, 5 °C for 8 h, and 10 °C for 4 h) and a short photoperiod (30–60% RH, 8: 16 h L: D). All of the plants were placed into a 60-mesh net box (60 cm in length, 30 cm in width, 30 cm in height). The clean virgin adult psyllids were released (n =  300), and 15 psyllids were examined every 7 days. Meanwhile, the control treatment was continuously reared in “suitable” conditions.

#### Dismount induction

On 8 March 2014, the psyllids were collected from the experimental field and then 2 adult psyllids (1 female and 1 male) were released onto one seedling 2 days later (n =  50). Each seedling was isolated with a transparent plastic pipe (15-cm high, 6-cm diameter with the top sealed with a 60-mesh net) and reared in climate-controlled boxes (25 °C, 30–60% RH, 16: 8 h L: D). The galls on the leaves caused by *A. pallida* were examined 5 days later.

### Morphological observations and measurements

The specimens of the gall mite *A. pallida* and its carrier *B. gobica* and the rust mite *A. lycii* used for scanning electron microscope (SEM) and morphological measurements were collected at the experimental field. The protogynes (non-phoretic stage) of *A. pallida* were collected from the galls of the host plant *L. barbarum*, and the protogynes of *A. lycii* were collected from the infected leaves on 28 July 2013. The deutogynes (phoretic stage) of *A. pallida* were collected on 08 January 2014 from the insect carrier *B. gobica*, and the deutogynes of *A. lycii* were collected on 12 March 2014 from their hibernation sites (bark, branches and buds). All of the morphological observations and measurements were conducted at the Institute of Medicinal Plant Development in Beijing, China.

Slides were mounted using Keifer’s F-medium and modified Berlese medium^47^, and the specimens were measured based on the methods outlined by de Lillo^48^. The specimens were examined with a Leica DM2500 (Leica Microsystems, Wetzlar, Germany) research microscope with phase contrast, and photographs of the slide-mounted mites were taken using the same microscope connected to a computer using Leica LAS image analysis software. The terminology used to describe the eriophyoid morphology and classification follows that of Lindquist^25^. For the SEM studies, the live eriophyoid specimens were prepared following the methods used for fresh eriophyoid mites, and the adult psyllids were routinely processed based on the “acrolein method”, according to Alberti and Nuzzaci^49^. The prepared samples were fastened onto stubs with double-sided tape and then coated with gold for 180 s at 30 mA in a JFC-1600 ion sputter (JEOL). They were then observed and photographed in a JSM-6510LV SEM (JEOL), and the SEM images of the phoretic mites were colorized in Photoshop CS5 (Adobe Systems). Four morphological characters (proterosoma length, hysterosoma length, body width and microtubercle length), which represent the main differences between the protogynes and the deutogynes^50^, were analysed, although there may be some other characteristics that differ slightly^50^. The length of the microtubercle (n =  200) was determined based on SEM, and the others characteristics were measured from slide images (n =  15 per character). The differences between the protogynes and deutogynes of *A. pallida* were evaluated statistically using a t-test to compare means in SPSS 20.0 software (IBM, Chicago, IL), and the values were reported as the mean ±  SE.

Additionally, to confirm whether the mites could be phoretic on other psyllid species with similar structures or other arthropods with different structures during the loading period (October~November), the psyllid *Euphalerus robinae* Shinji was collected from its host plant *Gleditsia japonica* Miq. near the experimental site on 10 October 2014, and then 60 adult psyllids were released into a 60-mesh net over a branch with phoretic mites. Then, the phoretic rate of *A. pallida* on *E. robinae* was investigated 3 days later. On 15 October 2014, other arthropods (ants: *Camponotus* sp.; aphids: *Aphis* sp.; ladybugs: *Propylaea japonica* Thunb.; stinkbugs: *Adelphocoris fasciaticollis* Reuter; and beetles: *Lema decempunctata* Gebler; n =  30 per species) that shared the same habitat with *A. pallida* were collected from *L. barbarum* at the experimental site. The numbers of phoretic *A. pallida* on *E. robinae* or other arthropods were examined under a Leica M205C stereomicroscope.

## Additional Information

**How to cite this article**: Liu, S. *et al.* Seasonal phoresy as an overwintering strategy of a phytophagous mite. *Sci. Rep.* **6**, 25483; doi: 10.1038/srep25483 (2016).

## Supplementary Material

Supplementary Information

## Figures and Tables

**Table 1:** Measurement of *A. pallida* protogynes and deutogynes.

Characteristic	Protogynes (μm)		Deutogynes (μm)	
	Mean ± SD	Min–Max	Mean ± SD	Min–Max
Proterosoma length	42.5 ± 3.51a	38.1–51.81	41.26 ± 1.56a	38.74–43.28
Hysterosoma length	207.27 ± 22.45A	170.28–239.87	128.34 ± 14.55B	102.48–154.15
Body width	67.56 ± 11.1A	50.31–82.21	47.26 ± 3.49B	39.96–53.57
Microtubercle length	1.02 ± 0.17A	0.64–1.47	1.59 ± 0.18B	1.23–2.06

The length of the microtubercle (n =  120) was determined based on SEM, and other (n =  15) measurements were made from slide images. Uppercase letters indicate a significant inter-group difference at the 1% level, and lowercase letters indicate a significant intra-group difference at the 5% level.
