# Supplementary material for: Seasonal phoresy as an overwintering strategy of a phytophagous mite
Source: Sci Rep. 2016 May 6;6:25483. doi: 10.1038/srep25483 (PMC4858688; doi:10.1038/srep25483)
Supplement: Supplementary Information [file srep25483-s1.pdf]

Seasonal phoresy as an overwintering strategy of a phytophagous mite

Sai Liu, Jianling Li, Kun Guo, Haili Qiao, Rong Xu, Jianmin Chen, Changqing Xu*, Jun Chen*

Figure S1

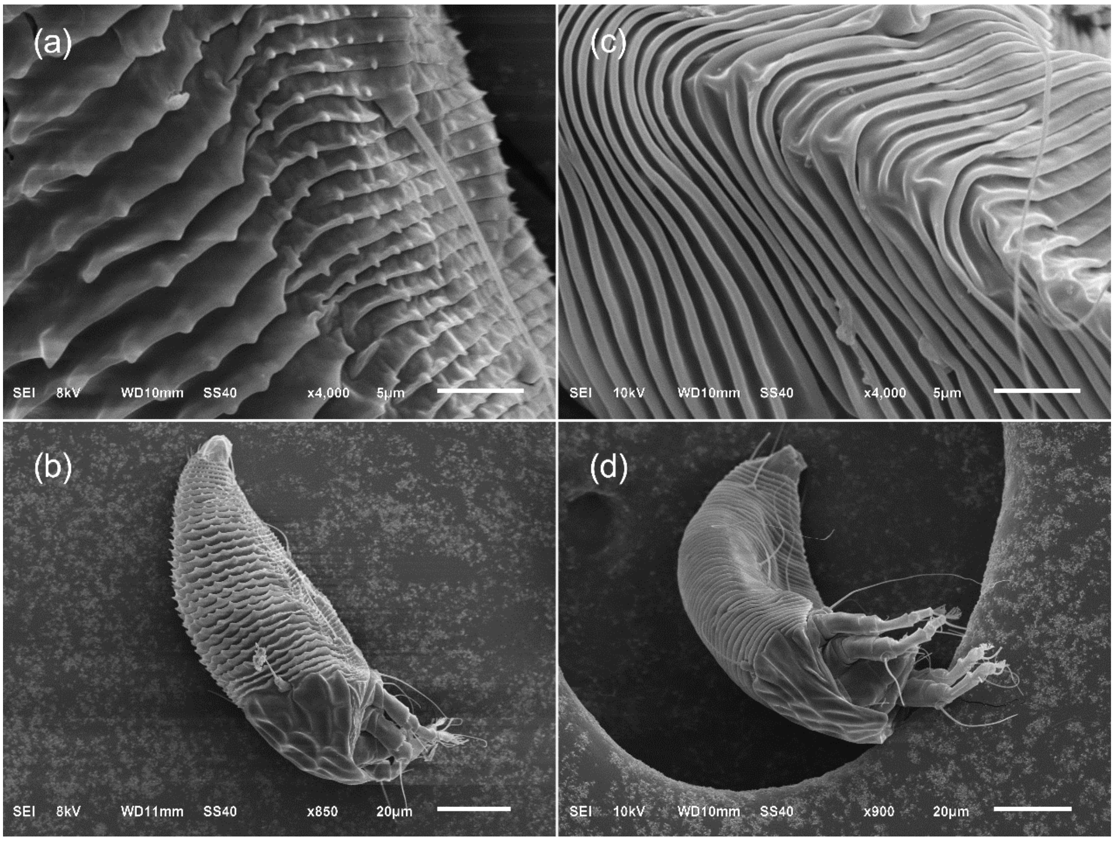

Figure S1. The microtubercles and slide views of the protogynes of *A. lycii* (**a**) (**b**) in summer and the deutogynes (**c**) (**d**) in winter. The deutogynes have significantly reduced microtuberculation.
